# Systemic corticosteroids in acute exacerbation of COPD: a meta-analysis of controlled studies with emphasis on ICU patients

**DOI:** 10.1186/s13613-014-0032-x

**Published:** 2014-10-26

**Authors:** Fekri Abroug, Islem Ouanes, Sarra Abroug, Fahmi Dachraoui, Saoussen Ben Abdallah, Zeineb Hammouda, Lamia Ouanes-Besbes

**Affiliations:** 1 ICU, Centre Hospitalier Universitaire Fatouma Bourguiba, Monastir 5000, Tunisia; 2 Research Lab LR12SP15, Centre Hospitalier Universitaire Fatouma Bourguiba, Monastir 5000, Tunisia

**Keywords:** COPD, Exacerbation, Corticosteroids, Critical care

## Abstract

Guidelines on systemic corticosteroids in chronic obstructive pulmonary disease (COPD) exacerbation rely on studies that excluded patients requiring ventilatory support. Recent publication of studies including ICU patients allows estimation of the level of evidence overall and in patients admitted to the ICU. We included RCTs evaluating the efficacy and safety of systemic corticosteroids in COPD exacerbation, compared to placebo or standard treatment. The effect size on treatment success was computed by a random effects model overall and in subgroups of non-ICU and ICU patients. Effects on mortality and on the rate of adverse effects of corticosteroids were also computed. Twelve RCTs (including 1,331 patients) were included. Pooled analysis showed a statistically significant increase in the treatment success rate when using systemic corticosteroids: odds ratio (OR) = 1.72, 95% confidence interval (CI) = 1.15 to 2.57; *p* = 0.01. Subgroup analysis showed different patterns of effect in ICU and non-ICU subpopulations: a non-significant difference of effect in the subgroup of ICU patients (OR = 1.34, 95% CI = 0.61 to 2.95; *p* = 0.46), whereas in the non-ICU patients, the effect was significant (OR = 1.87, 95% CI = 1.18 to 2.99; *p* = 0.01; *p* for interaction = 0.72). Among ICU patients, there was no difference in the success whether patients were ventilated with tracheal intubation (OR = 1.85, 95% CI = 0.14 to 23.34; *p* = 0.63) or with non-invasive ventilation (OR = 4.88, 95% CI = 0.31 to 75.81; *p* = 0.25). Overall, there was no difference in the mortality rate between the steroid-treated group and controls: OR = 1.07, 95% CI = 0.67 to 1.71; *p* = 0.77. The rate of adverse events increased significantly with corticosteroid administration (OR = 2.36, 95% CI = 1.67 to 3.33; *p* < 0.0001). In particular, treatment with systemic corticosteroids significantly increased the risk of hyperglycemic episodes requiring initiation or alteration of insulin therapy (OR = 2.96, 95% CI = 1.69 to 5; *p* < 0.0001). We found corticosteroids to be beneficial in the whole population (non-critically ill and critically ill patients) in terms of treatment success rate. However, subgroup analysis showed that this effect of corticosteroids was only observed in non-critically ill patients whereas critically ill patients derived no benefit from systemic corticosteroids regardless of the chosen ventilatory mode (invasive or non-invasive ventilation). Further analyses showed no effect on mortality of corticosteroids, but higher side effects, such as hyperglycemic episodes requiring the initiation or alteration of insulin therapy.

## Review

### Introduction

Chronic obstructive pulmonary disease (COPD) is a common disease characterized by progressive and incompletely reversible chronic airflow obstruction associated with an exaggerated inflammatory response to bacteria, viruses, or pollutants [1]. COPD has become the third leading cause of mortality worldwide, with attributable yearly costs of no less than 50 billion $ in the United States [2]-[4]. Approximately 60% of these expenditures are dedicated to the management of exacerbations of COPD [4]. COPD exacerbation is defined as an acute event in which there is impairment of respiratory symptoms beyond the level of daily changes requiring an alteration of the usual treatment [1]. Each patient experiences a mean of two exacerbations per year, which are linked to a steady decline in lung function, quality of life, and life expectancy [5]-[7]. Exacerbation of COPD is associated with an accentuation of systemic and local inflammation [8]-[11]. In combination with inhaled bronchodilators, systemic corticosteroids are the cornerstone of the first-line treatment of exacerbation of COPD and are strongly recommended by current guidelines [12]-[17]. Yet, the optimal systemic corticosteroid formulation (e.g., methylprednisolone or prednisone), daily dose (moderate vs high), route of administration (oral or intravenous), and treatment duration (5 to 15 days) are unclear [16],[18]-[22]. This drug combination improves symptoms and lung function and reduces the duration of hospitalization [23]. However, systemic corticosteroids do not affect mortality rate related to COPD exacerbation and are associated with known adverse effects including increased frequency of hyperglycemic episodes (up to five times compared to untreated patients) [15],[16].

In addition, most studies supporting corticosteroid treatment in COPD exacerbation have been conducted outside the ICU and have deliberately excluded patients with severe exacerbation requiring ventilatory support in the ICU, making hazardous extrapolation of their results to patients admitted to the ICU especially when we refer to steroids’ side effects that are peculiar to critically ill patients: ICU-related neuromyopathy, interference with sepsis evolution, hyperglycemic accidents and their impact on the length of ventilatory support, etc. [24],[25].

Two recent studies dealing specifically with severe COPD exacerbation requiring ventilatory support yielded conflicting results. Nevertheless, despite being underpowered, these newly published studies on ICU patients shed light on the level of evidence surrounding corticosteroid treatment in ICU patients with COPD exacerbation.

The aim of this systematic review with meta-analysis of the literature is to measure the level of scientific evidence on systemic corticosteroid treatment in the case of exacerbation of COPD in general, with particular emphasis on critically ill patients admitted to the ICU, in the light of recent publications on this issue.

### Materials and methods

#### Search strategy and study selection

Pertinent studies were searched in MEDLINE, EMBASE, CINAHL, CENTRAL, and Science Citation Index for RCTs published up to 1 June 2014 with the following MeSH terms: [‘adrenal cortex hormone’ or ‘steroid’ or ‘corticosteroid’ or ‘hydrocortisone’ or ‘prednisone’ or ‘prednisolone’ or ‘methylprednisolone’ or ‘dexamethasone’] AND [‘COPD’ or ‘chronic obstructive pulmonary disease’ or ‘lung disease, obstructive NOT asthma’] AND [‘exacerbation’ or ‘AECOPD’ or ‘emergency’]. We have also looked for studies in other data sources such as reference reports, journal articles, and abstracts of conferences and conducted a manual search in journals.

#### Study selection

We included all randomized controlled clinical trials designed to evaluate the efficacy and safety of systemic corticosteroids, whether administered orally or parenterally, during acute exacerbations of chronic obstructive pulmonary disease (AECOPD), compared to placebo or standard treatment. Other treatments (e.g., bronchodilators, antibiotics) administered concomitantly had to be comparable between the two arms of studies. Included studies involved patients seen in an outpatient setting, patients in emergency consultation, or patients hospitalized in the pulmonology ward or intensive care units.

Patients included in these studies were adults aged 18 and older with COPD, with recent acute exacerbation identified clinically by the modification of functional signs of COPD according to the Anthonisen criteria [26]: worsening of recent dyspnea, cough, and change in the characteristics of sputum consisting in the increase in sputum volume or its appearance.

#### Data extraction and study characteristics

Two independent evaluators (LOB, IO) selected studies according to the inclusion criteria and extracted the intervention in each study, the type of patients, the location of the study, and the criteria for primary and secondary endpoints. Disagreements were resolved by consensus. The quality of included studies was assessed by the Jadad scale [27], which assigns a score ranging from 0 to 5.

#### Data synthesis

In this meta-analysis, the primary endpoint was treatment success, which had different definitions in the included studies. In outpatients and in those admitted to general wards, treatment was deemed successful on the basis of clinical improvement that was assessed by a questionnaire and/or improvement in forced expiratory volume in 1 s (FEV1), improvement in blood gases, or reduction in the duration of hospitalization. In studies dealing with ICU patients under ventilatory support, successful treatment was defined as the lack of need for intubation in patients treated with non-invasively ventilated (NIV) patients and reduced mortality rate in invasively (intubated) ventilated patients.

Secondary outcomes included mortality whenever reported for both groups and the rate of adverse effects of corticosteroids. For the study purpose, we considered the following: overall side effects, hyperglycemic episodes requiring the initiation or changing of current insulin therapy, psychiatric disorders, infections occurring during hospitalization, hypertensive episodes, and gastrointestinal bleeding.

#### Statistical analysis

The meta-analysis was conducted using the Comprehensive Meta-Analysis (CMA) program V2 software (Biostat, Englewood, NJ, USA). This meta-analysis was conducted in accordance with the recommendations of the Cochrane group and the PRISMA guidelines.

For binary outcomes (treatment success, mortality, and adverse effects of systemic corticosteroids), we reported the outcome estimates as odds ratios (ORs) with 95% confidence intervals (CIs). Statistical significance was set at *p* <0.05 for hypothesis testing and *p* <0.1 for heterogeneity testing. We measured heterogeneity and expressed it as *I*^2^, with suggested thresholds for low (*I*^2^ = 25% to 49%), moderate (*I*^2^ = 50% to 74%), and high (*I*^2^ ≥ 75%) values. We used a random effects model which merges the studies and provides an assessment of more conservative treatment effect when heterogeneity is present or suspected. We made this choice because of the obvious heterogeneity of the studies included in the analysis.

To assess publication bias, we visually examined the funnel plot for mortality and performed the Egger test of the intercept which uses precision to predict the standardized effect. All statistical tests were two-sided.

Two subgroup analyses were performed in this meta-analysis: the first one compared the effect of systemic corticosteroids in patients who were admitted to the ICU compared to that in patients treated elsewhere. The second subgroup analysis compared patients from the ICU subgroup, according to the ventilatory mode: invasive or non-invasive ventilation.

### Results

#### Search results and trial characteristics

**Figure 1:**
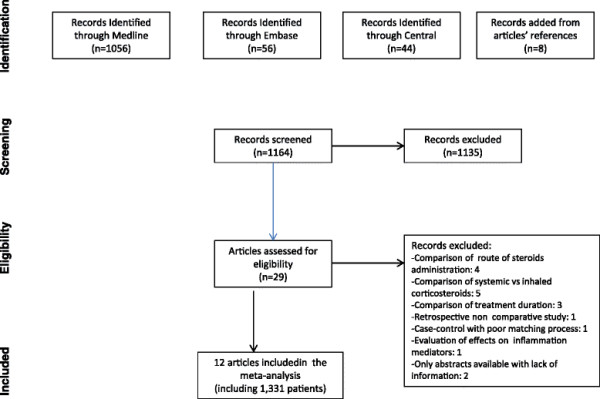
Flow chart of the meta-analysis.

Literature search initially identified 1,156 citations; 5 items were added after consulting the bibliography of previously published meta-analyses and journals. Among these studies, 29 were identified as related to the use of corticosteroids during AECOPD. Ultimately, 17 studies were excluded, and 12 were included with a total of 1,331 patients [23],[28]-[38]. The selection process is illustrated by a flow chart (Figure 1). The main clinical characteristics of included studies are depicted in Table 1.

**Table 1:** Patients characteristics and steroid regimen in the studies included in the meta-analysis

**Study and publication year**	**Setting**	**Exacerbation criteria**	**Mean age (SD)**	**Patient number**		**Intervention**	**Duration (days)**	**Endpoint and success criteria**	**Jadad score**
				**Systemic corticosteroids (*n*/*N*)**	**Controls (*n*/*N*)**				
Albert 1980 [29]	Pulmonology ward	Clinical and pulmonary function	61.5 ± 9.5	22/44	22/44	Methylprednisolone IV 0.5 mg/kg/6 h × 72 h	3	Improvement in lung function	5
Emerman 1989 [28]	ED	Clinical and pulmonary function	64.0 ± 7.8	52/96	44/96	Methylprednisolone IV 100 mg single injection	1	Improvement in lung function; no need for hospitalization	5
Bullard 1996 [30]	ED/pulmonology ward	Clinical and pulmonary function	66.0 ± 10.9	60/113	53/113	Hydrocortisone IV 100 mg/4 h × 4 days or until discharge, then prednisolone PO 40 mg/day × 4 days	8	Improvement in FEV1 at 6 h; no relapse or ED visit	4
Thompson 1996 [31]	Ambulatory	Clinical	67.8 ± 8.6	13/27	14/27	Prednisone PO 60 mg/day × 3 days followed by 40 mg/day × 3 days, then 20 mg/day × 3 days	9	Improvement in FEV1 at days 1, 3, and 10; improved blood gases and clinical symptoms	5
Wood-Baker 1997 [32]	Pulmonology ward	Clinical	72 ± 6.3	12/38	13/38	(1) Prednisolone PO 2.5 mg/kg/day × 3 days, then placebo × 11 days	3	Improvement in lung function, 6-min walk test, hospitalization duration, improvement in clinical symptoms	5
				13/38		(2) Prednisolone PO 0.6 mg/kg/day × 7 days, then 0.3 mg/kg/day × 7 days	14	FEV1 at day 1 and at 6 weeks, hospitalization duration, clinical improvement	
Davies 1999 [33]	Pulmonology ward	Clinical and pulmonary function. Exacerbation without acidosis	67.3 ± 8.4	29/56	27/56	Prednisone PO 30 mg/day × 14 days	14	Elapsed time until treatment failure, improvement in FEV1, hospitalization duration	5
Niewoehner 1999 [23]	Pulmonology ward	Clinical and pulmonary function	67.7 ± 9.3	80/271	111/271	(1) Methylprednisolone IV 125 mg/6 h × 72 h followed by prednisone PO 60 mg/day with slow tapering for 54 days	57	Improvement in FEV1 and blood gases at day 3, clinical improvement, hospitalization duration	5
				80/271		(2) Methylprednisolone IV 125 mg/6 h × 72 h followed by prednisone PO 60 mg/day with slow tapering for 12 days	15	Lack of relapse or rehospitalization, improvement in FEV1, clinical improvement and improvement in life quality at day 10	
Maltais 2002 [34]	Pulmonology ward	Clinical and pulmonary function	70.4 ± 8.3	62/128	66/128	Prednisone PO 30 mg/12 h × 3 days, then 40 mg/day × 7 days	10	Improvement in lung function, blood gases, and reduction in hospitalization duration	4
Aaron 2003 [35]	Ambulatory	Clinical	69.4 ± 10.8	74/147	73/147	Prednisone PO 40 mg/day × 10 days	10	I: no relapse or readmission, II: improvement in FEV1, clinical status, and quality of life at day 10	5
Chen 2008 [36]	Pulmonology ward	Clinical	71.6 ± 7.3	44/130	43/130	(1) Prednisone PO 30 mg/day × 7 days	7	Improvement in FEV1, blood gases, hospitalization duration	5
				43/130		(2) Prednisone PO 30 mg/day × 10 days, then 15 mg/day × 4 days	14		
Alia 2011 [37]	ICU	Clinical	68.4 ± 10.2	43/83	40/83	Methylprednisolone IV 0.5 mg/kg/6 h × 3 days, then 0.5/kg/12 h × 3 days followed by 0.5 mg/kg/day × 4 days	10	Mechanical ventilation duration, ICU stay, and intubation rate	5
Abroug 2014 [38]	ICU	Clinical	69.0 ± 6	111/217	106/217	Prednisone 1 mg/kg/day × 10 days maximum or until discharge	10	Non-invasive ventilation success, ICU mortality in intubated patients	3

R, randomization; B, blindness; L, lost to follow-up; SD, standard deviation; *n*, patients in the study arm; *N*, total sample size.

All studies were randomized, controlled, double-blind studies except that of Abroug et al. [38] which was a randomized, controlled, open-label study including critically ill patients. All studies had a Jadad score ≥3. Eighty-two percent of included patients (1,092/1,331) were male, with a mean age of 66 ± 14 years. Smoking was reported in seven studies. Overall, 47% of the included patients were smoking more than ten pack-years.

The definition of AECOPD was based on clinical symptoms or on the results of respiratory evaluation.

There was no difference in demographic characteristics between the groups receiving systemic corticosteroids and the control groups regarding main clinical characteristics (Table 1).

The administered corticosteroid regimen varied among studies with different formulations, routes of administration, and duration of treatment which ranged from 1 to 57 days. The duration of patient follow-up and wards of inclusion and management of patients were also variable (outpatients, emergency department, respiratory ward, or ICU) (Table 1). Criteria defining the success of corticosteroid treatment were also variable (Table 1): this encompassed the reduction in the length of hospital stay, increased FEV in non-ICU patients, and decreased intubation rate and mortality in ICU patients.

Adverse effects related to corticosteroid treatment have been reported in eight studies with emphasis on hyperglycemia, infections, hypertension, gastrointestinal bleeding, and psychiatric symptoms.

#### Data analysis

Comparison of systemic corticosteroids’ efficacy involved 722 patients who received treatment and 609 controls.

##### Primary endpoint: treatment success

**Figure 2:**
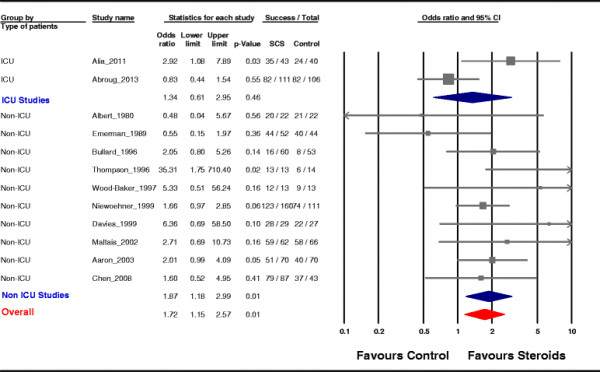
**Pooled subgroup analysis.** Effects of corticosteroid treatment on the success rate inferred from all included studies and separately for the groups of studies on ICU patients and on non-ICU patients. Gray squares represent odds ratios (ORs) in individual trials with the size proportional to the weight of the study. The 95% confidence intervals (CIs) for individual trials are denoted by lines. The pooled subgroup estimate of the effect is represented by the blue diamond (with a width proportional to the confidence interval), and the combined overall effect is represented by the red diamond. The *I*^2^ test for heterogeneity was moderate overall (*I*^2^ = 36%), high in the analysis involving critically ill patients (*I*^2^ = 77.4%), and low in studies including non-ICU patients (*I*^2^ = 17.4%). The *z* test for interaction between subgroups was 0.36 (*p* = 0.72). The meta-analysis is performed by a random effects model.

**Figure 3:**
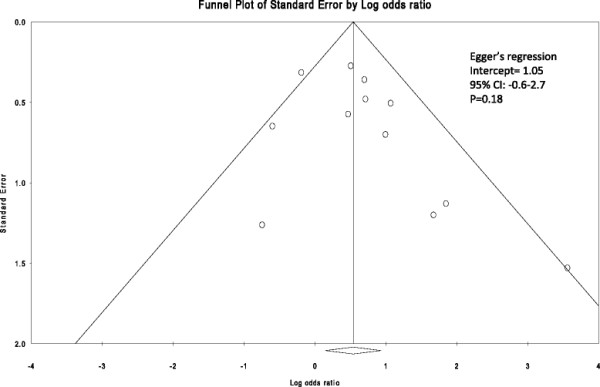
**Funnel plot of SE by odds ratio.** The Egger test was non-significant (regression intercept = 1.05, *p* = 0.18).

Pooled analysis shows a statistically significant increase in the treatment success rate when using systemic corticosteroids: OR = 1.72, 95% CI = 1.15 to 2.57; *p* = 0.01 (Figure 2). Overall, heterogeneity was low (*I*^2^ = 36%). There was no obvious publication bias detected by visual inspection of the ‘funnel plot’ (Figure 3). The Egger test was also non-significant (regression intercept = 1.05, *p* = 0.18).

Subgroup analysis which compared studies that included ICU patients vs studies that excluded ICU patients (non-ICU patients) showed different patterns of effect in these two subpopulations: the effect of steroid administration was not statistically significant in the subgroup of ICU patients (OR = 1.34, 95% CI = 0.61 to 2.95; *p* = 0.46), whereas in the subgroup of non-ICU patients, the effect was significant and similar to what has been observed in the entire analysis (OR = 1.87, 95% CI = 1.18 to 2.99; *p* = 0.01; *z* test for interaction = 0.36, *p* = 0.72) (Figure 2).

Heterogeneity was high in the analysis involving critically ill patients (*I*^2^ = 77.4%), while it was low in studies including non-ICU patients (*I*^2^ = 17.4%).

In order to explore the high heterogeneity observed in studies that included ICU patients, we compared the outcome in these patients according to the mechanical ventilation modality (invasive vs non-invasive ventilation). There was no difference in the effect thereof. The odds ratio was 1.85, 95% CI = 0.14 to 23.34; *p* = 0.63, for the group of patients ventilated invasively through an endotracheal tube and 4.88, 95% CI = 0.31 to 75.81; *p* = 0.25, for the NIV group.

##### Secondary criteria

**Figure 4:**
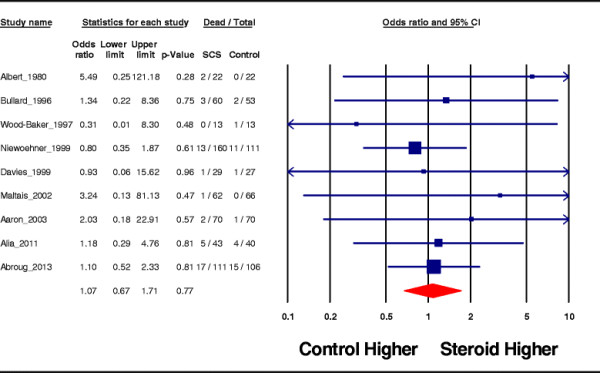
Effect on mortality.

Mortality was analyzed in nine studies. Overall, the mortality rates were not statistically different between the steroid-treated group and controls: OR = 1.07, 95% CI = 0.67 to 1.71; *p* = 0.77; *I*^2^ = 0% (Figure 4).

**Figure 5:**
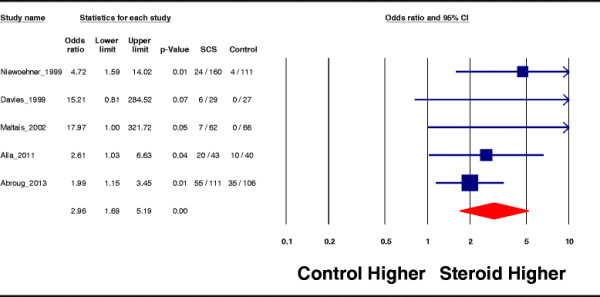
Hyperglycemic episodes.

For adverse effects, overall, seven studies reported the rate of adverse events linked to treatment by systemic corticosteroids and showed a significant increase in this risk: OR = 2.36, 95% CI = 1.67 to 3.33; *p* <0.0001; *I*^2^ = 3%. In particular, hyperglycemic episodes requiring initiation or alteration of insulin therapy which were reported in five studies were increased by corticosteroid administration: OR = 2.96, 95% CI = 1.69 to 5; *p* <0.0001; *I*^2^ = 22.7% (Figure 5).

### Discussion

The current meta-analysis shows that overall systemic corticosteroid therapy increases the rate of success of the treatment of patients with COPD exacerbation. Stratified analysis according to the exacerbation severity (severe hypercapnic exacerbation requiring ventilatory support in the ICU, compared to exacerbations not requiring ventilatory support) showed that this effect of corticosteroids was only observed in non-critically ill patients whereas critically ill patients derived no benefit from systemic corticosteroids regardless of the chosen ventilatory mode (invasive or non-invasive ventilation). Further analyses showed no effect of corticosteroids on mortality, but higher side effects, such as hyperglycemic episodes requiring the initiation or alteration of insulin therapy.

Current guidelines strongly recommend the administration of systemic steroids to patients experiencing COPD exacerbation whatever its severity. In fact, most of the evidence stems from studies in which patients were treated on an outpatient basis or hospitalized outside ICUs. The most severe patients who are usually admitted to ICUs and receive ventilatory support were usually excluded. In addition, despite the lack of important statistical heterogeneity, the global analysis we have conducted suffers probably from obvious clinical heterogeneity of included studies. This heterogeneity stems from substantial differences in the steroid regimen applied in these studies, the studies’ setting (emergency department, pulmonology ward, or ICU), differences in success criteria, and the criteria used for COPD and exacerbation. All these discrepancies might indeed introduce bias to overall inferences from these studies. Substantial variation in corticosteroid regimen (daily dose, treatment duration, route of administration) might have impacted *per se* the rate of treatment success provided that the chosen regimen is close to or far from the ideal corticosteroid regimen, which is not validated so far. In addition, pooling the results of studies conducted in patients with various levels of severity (wards or ICU patients) did not take into account the relationship between baseline severity and success rate. Moreover, Bullard et al. and Chen et al. did not specifically exclude asthmatics suggesting that if these patients have actually been included, they could have better responses to steroid treatment than the remaining study patients [30],[36].

Moreover, the extrapolation to ICU patients of what was observed in less severe patients should not be straightforward since administration of systemic steroids to ICU patients should outweigh the specific harm incurred by such treatment in critically ill patients (neuromyopathy, facilitation and worsening of sepsis, etc.). Moreover, beneficial effects of corticosteroids reported so far were inferred from the impact on outcomes which should not be regarded as patient-centered. Indeed, outcomes that were positively impacted by corticosteroid treatment (lung function, hypoxemia, relapse rate) are not those that one expects to alter in life-threatening exacerbations. Outcomes such as non-invasive ventilation failure, ICU mortality, MV duration, and length of ICU stay are indeed more relevant to ICU patients.

As a matter of fact, we are today in a situation where even the ‘equipoise’ surrounding the use of systemic steroids in COPD exacerbation is questioned [39]. This is suggested by reports from registries reflecting the real-world practice. Lindenauer et al. reported that no less than 76% patients treated for COPD exacerbation outside the ICU received either a high (92%) or a moderate (8%) dose of systemic steroids [20]. Examining the same database, but focusing on patients who were excluded from the Lindenauer study, namely patients with severe COPD exacerbation who were admitted to the ICU, Kiser et al. report that only 6% were not treated by systemic steroids while 64% of the remaining received a very high dose of steroids (i.e., >240 mg/day methylprednisolone equivalent) [19]. Both analyses clearly show that there is very little equipoise, if any, about the use of systemic corticosteroids in COPD exacerbation whether requiring mechanical ventilation or not. Hopefully, the scientific evidence concerning specifically patients with severe COPD exacerbation requiring ventilatory support became recently available. Alia et al. and Abroug et al. published two RCTs including ICU patients [37],[38]. The first one was a positive Spanish multicenter, randomized, double-blinded study that included 83 patients, showing a 1-day reduction in the duration of ICU hospitalization [37]. The second study was an open-label study conducted in two ICUs in Tunisia with the aim of evaluating the effect of a 10-day prednisone course on ICU mortality [38]. This study was a negative one, failing at demonstrating any beneficial effect of steroid treatment on ICU mortality, length of mechanical ventilation, and ICU stay. We cannot readily account for these conflicting results, but there were substantial differences in the use of non-invasive ventilation. NIV was more frequently applied in the Abroug et al. study (76% vs 44% in the Alia et al. study) [37],[38]. Moreover, despite fairly similar rates of NIV failure overall, these were not distributed similarly in the studies’ arms. While Abroug et al. reported similar failure rates in both study groups (16% and 13% in prednisone-treated and control groups, respectively), Alia et al. reported a striking difference in NIV failure rate which was 0% in the steroid treated group and 37% in the control group [37],[38]. Both rates reported by Alia et al. seem overly shifted from rates usually reported in the literature [40].

Noteworthy, both studies were also stopped before completion of the inclusion of the planned sample size (25% and 73% of planned samples, respectively). Alia et al. included 83 out of the 198 patients planned to show a 1-day reduction in mechanical ventilation duration [37]. On the other hand, out of 300 patients required to demonstrate a reduction by 12% in the absolute rate of ICU mortality, Abroug et al. eventually included only 217 patients [38]. This was due principally to the fact that a large proportion of patients considered for inclusion (up to half in each study) was already treated by corticosteroids at ICU admission. Indeed, the administration of corticosteroids prior to hospitalization was so widespread that many patients were not eligible for both RCTs, illustrating the difficulties in completing such studies in the ICU. Despite apparently conflicting results, both studies concluded that it would take up to 2,000 patients to uncover an impact on hard outcomes such as ICU mortality, NIV failure, etc. So how can we answer such questions in ICU patients in order to reach a sufficient level of evidence? Systematic reviews and meta-analytic techniques offer in this respect interesting resources, especially through the stratification of the analysis according to the type of patients whether treated in or outside the ICU. The fact that recent publications on the issue yielded conflicting results makes it very useful to examine the trend from the pooled effects through meta-analytic techniques. Previous meta-analyses included only primary studies that were conducted in non-ICU patients. Although systemic corticosteroids had no effect on mortality, these meta-analyses showed that systemic corticosteroids yielded a statistically significant reduction in treatment failure and/or relapse, improvement in FEV1, and oxygenation [12],[13],[15]-[17]. The current meta-analysis evaluated specifically the effect of systemic corticosteroids in severe COPD exacerbation requiring ventilatory support. While the global analysis showed a favorable effect of systemic corticosteroid therapy, subgroup analysis in the subset of studies that included only patients requiring ventilatory support reveals that available data synthesis did not show any beneficial effect from systemic corticosteroid treatment regarding either the rate of treatment success or that of ICU mortality. Still, there are a 2.4-fold increase in the adverse effects of steroid treatment overall and a threefold increase in the hyperglycemic episodes. This provides an additional argument which obviously weighs negatively in the risk-benefit balance surrounding this treatment. Nevertheless, absence of evidence of benefit should not be regarded as evidence of the absence of corticosteroids’ benefit. The lack of a significant test for interaction means that our finding does not rule out the possibility that this difference could be due to chance and does not ascertain that systemic steroids have indeed an opposite effect in ICU and non-ICU patients, especially with the finding of inconsistent results in ICU studies [41]. Moreover, the examination of the boundaries around the estimation point in the present meta-analysis rules out neither a deleterious effect of corticosteroids (39% risk of a greater failure rate) nor an increase by threefold in the success rate of corticosteroid treatment. A large study including ICU patients is urgently needed in order to draw a definitive conclusion in this subgroup of patients.

It may seem surprising that we advocate larger randomized controlled trials on corticosteroids in this population when the two available studies in critically ill patients were early stopped because of recruitment difficulties. We believe that the situation of equipoise prevailing now is different from that prevailing before the publication of the two RCTS on ICU patients and the combined analysis made here. We should no longer ignore the new evidence warning against potential deleterious effects of administering systemic corticosteroids in COPD patients requiring ventilatory support [39].

## Conclusions

This meta-analysis confirms that the rate of treatment success increases with systemic corticosteroids in comparison to usual care in COPD exacerbation. Recent publication of two RCTs dealing specifically with severe exacerbations requiring ventilatory support allowed a stratified analysis according to the necessity of ventilatory support in the ICU. For patients treated in the ICU, there is no sufficient level of scientific evidence enabling a strong recommendation. Current data cannot exclude both possibilities: efficacy or inefficacy. Larger studies targeting such patients are still needed.

## Competing interests

The authors declare that they have no competing interests.

## Authors’ contributions

FA, IO, and SA conducted the literature searches, selected the studies, extracted the data, and assessed the study quality. FA prepared the initial and subsequent drafts of the manuscript. FD, SBA, ZH, and LOB screened the abstracts, selected the studies meeting the inclusion criteria, extracted the data, and assessed the study quality. FA and SA carried out the statistical analyses with input from all other authors. All authors read, revised, and approved the final manuscript.
